# Epidemiology and natural history of atopic diseases

**DOI:** 10.3402/ecrj.v2.24642

**Published:** 2015-03-24

**Authors:** Simon F. Thomsen

**Affiliations:** Department of Dermatology, Bispebjerg Hospital, Copenhagen, Denmark

**Keywords:** asthma, atopic diseases, natural history, allergic march, epidemiology

## Abstract

The atopic diseases – atopic dermatitis, asthma, and hay fever – pose a great burden to the individual and society, not least, since these diseases have reached epidemic proportions during the past decades in industrialized and, more recently, in developing countries. Whereas the prevalence of the atopic diseases now seems to have reached a plateau in many Western countries, they are still on the increase in the developing world. This emphasizes continuing research aimed at identifying the causes, risk factors, and natural history of these diseases. Herein, the fundamental aspects of the natural history and epidemiology of the atopic diseases are reviewed.

The word *atopy* (Greek: *atopia*, out of place) refers to an inherited tendency to produce immunoglobulin E (IgE) antibodies in response to small amounts of common environmental proteins such as pollen, house dust mite, and food allergens. The presence of atopy in an individual is associated with an increased risk of developing one or more of the atopic diseases – atopic dermatitis, asthma, and allergic rhinoconjunctivitis/hay fever (and food allergy). However, atopy can be present in the form of asymptomatic sensitization to one or more allergens, which means that an individual with confirmed allergic sensitization does not exhibit clinical allergy. Conversely, an atopic disease can be present in a non-sensitized individual (e.g. in non-atopic asthma). Sensitization is common in the atopic diseases of childhood and is less frequent in adults with atopic disease, particularly in those with adult-onset atopic disease (1). Asymptomatic sensitization to aeroallergens, however, is a strong predictor for future development of allergic symptoms, while allergic symptoms in non-sensitized subjects are a much lower risk factor for subsequent sensitization (2).

## The allergic march

*The allergic march* refers to the natural history of atopic disorders. The allergic march concerns the development of atopic dermatitis and concomitant sensitization to food and aeroallergens in early childhood, progressing to asthma and allergic rhinitis in later childhood or adult life (3).

Typically, the child develops atopic dermatitis in the first months of life accompanied by sensitization to cow's milk, egg, or peanut, and sometimes also vomiting, diarrhoea, or anaphylaxis in relation to ingestion of these foods beginning around the age of 6–12 months. This is followed by sensitization to indoor allergens such as house dust mite, cockroach, and furred pets. Within the first 2 years of life the child develops recurrent episodes of wheezing, mostly in conjunction with viral respiratory tract infections such as *respiratory syncytial virus* and *rhinovirus* (4). After this age wheezing episodes become more frequent and start to occur in between infections, requiring continuous treatment with asthma medication, and now can be said to have manifested asthma. Later in childhood, allergy to outdoor allergens develops and allergic rhinoconjunctivitis occurs in relation to exposure to grass and tree pollen. At the same time, eczema and sensitisations to food wane, but cross-reactions to nuts and fresh fruits and vegetables may develop and give rise to oral allergic manifestations. Despite the teenage years being a time when asthma symptoms may disappear or become less pronounced, after some symptom-free years, skin and respiratory symptoms return in a subset. In young and middle adulthood, respiratory and skin manifestations are more closely related to occupational exposures, lifestyle, and tobacco smoking, so that, for instance, hand eczema or asthma-chronic obstructive pulmonary disease overlap syndrome (ACOS) develops in relation to these exposures. In late adulthood, allergic symptoms generally become less frequent and tend to disappear but in some, new-onset allergy or asthma may develop in old age (5).

In childhood the incidence of atopic manifestations is typically higher among boys, but this tendency changes in adolescence and in adulthood, during which girls become more symptomatic (6). Although the skin sensitization occurring in atopic dermatitis appears to be the trigger for the subsequent development of the other allergic conditions, the progression is not uniform in all atopic children. Allergic manifestations can develop at any point in life. Many will experience only one or perhaps two atopic manifestations and the development of these can be interspaced by several years; therefore, it is not uncommon that an adult with ‘new-onset’ asthma is unable to remember whether he or she had asthma or eczema in childhood. In some children, the sequence of events is reversed so that asthma precedes the development of eczema, and sometimes symptoms occur simultaneously, rendering the age of onset of the different disorders indistinguishable. Accordingly, the severity of the atopic syndrome varies highly between affected individuals and the course of the disease depends on a dynamic interplay between many innate and triggering factors.

The discovery of filaggrin gene mutations as a predisposing factor for atopic dermatitis and subsequent asthma and sensitization in the context of eczema has redefined our view on the allergic march (7). The atopic diseases can now be viewed upon as causally related conditions – rather than sequentially occurring manifestations of the same underlying disease state – with atopic dermatitis and filaggrin mutations being a prerequisite for the development of the other atopic diseases, particularly asthma (8).

Only few prophylactic interventions have been shown to significantly influence the risk of developing atopic diseases in the long run. The advent of filaggrin gene mutations holds promise that progression of the allergic march from atopic dermatitis to asthma can be halted by treating the skin barrier defects in infants and very young children (9).

### Atopic dermatitis

Atopic dermatitis is primarily a disease of early childhood. About 20% of all children develop symptoms of atopic dermatitis at some point in their lives (10). Half of these develop symptoms within the first year of life with 95% experiencing onset below 5 years of age. The majority outgrows atopic dermatitis in childhood or early adolescence, but around 25% continue to have eczema into adulthood or experience a relapse of symptoms after some symptom-free years. Up to one fourth of subjects with moderate to severe atopic dermatitis in childhood will develop hand eczema to various degrees in adult life (11).

About 30% of all children with atopic dermatitis have food allergy. The allergens involved are typically cow's milk and egg with other foods also being common, for example, soy, wheat, and fruits. However, intake of foods is rarely the cause of exacerbations in atopic dermatitis and many patients with atopic dermatitis are sensitized to foods without this being involved in the activity of the disease.

The risk of other atopic diseases, primarily asthma and hay fever, is markedly increased in children with atopic dermatitis. A child with moderate to severe atopic dermatitis has a 50% risk of developing asthma, either concomitantly or in later life, whereas the risk of developing hay fever is as much as 75% (12).

### Food allergy

Many patients tend to confuse real food allergies with non-allergic food reactions, such as food intolerance, which gives an impression in the public that real IgE-mediated food allergy occurs more frequently than is the case.

More than 170 foods have been reported to cause IgE-mediated reactions, but the allergens most commonly involved are cow's milk, egg, nuts, fish, and shellfish (13). Food allergy typically develops in the order in which the individual is exposed to the specific food. Therefore, allergy to cow's milk develops in early childhood followed by allergy to egg (13). In older children, allergy to wheat and fresh fruit develops, and in adults, the most common food allergies relate to nuts and shellfish as well as food allergy due to cross-reactivity with pollen. Self-reported symptoms suggestive of allergy to these foods occur in about 10–15% of children and adults. However, the prevalence of confirmed food allergy is only about 3–5%. Food-induced anaphylaxis is a very serious event that occurs in less than 0.1% of the general population.

Most children with food allergy will eventually tolerate cow's milk, egg, soya, and wheat, but far fewer will eventually tolerate tree nuts and peanut. The time course of food allergy resolution in children varies according to the foodstuff and may occur as late as the teenage years. For example, in children diagnosed with egg allergy in early life, two thirds will be tolerant by school age and most with allergy to cow's milk will tolerate cow's milk in adolescence. Risk factors for persistence of egg allergy are a high initial level of egg serum IgE, the presence of other atopic diseases, and the presence of an allergy to another food. With avoidance diet, up to 15–20% of children will remain allergic and the severity of the reactions may increase with time. In these severe cases of egg allergy, it becomes more difficult to adhere to the avoidance diet, with a considerable decrease in patients’ quality of life (14). Induction of oral tolerance can be regarded as a therapeutic option for IgE-mediated egg allergy. Omalizumab (anti-IgE) might become a therapeutic option for food allergy, not only to prevent allergic reactions after a contact with egg, but also as a complementary treatment to oral tolerance induction for egg allergy, with the purpose of reducing adverse reactions (14). Administration of influenza vaccine to children with egg allergy is safe in children that do not manifest severe reactions after egg intake, and in children who tolerate cooked egg. The triple viral vaccine (MMR) can be given to egg-allergic children with no increased risk. Different medicinal products are formulated with egg proteins, and therefore should be avoided in children with egg allergy (14). In children, a drop in serum IgE levels over time is often a marker of the onset of tolerance to the food. In contrast, for some foods, the onset of allergy can occur in adult life, and the food allergy may persist despite a drop in IgE levels over time. A high initial level of serum IgE against a food is associated with a lower rate of resolution of clinical allergy over time. Food allergy that develops in adulthood has a much more unfavourable prognosis and, as a rule, will persist throughout life.

Food allergy is strongly associated with the occurrence of other atopic disorders. About 50% of all children with food allergy have atopic dermatitis, about 40% have asthma, and about 30% have allergic rhinitis (13).

### Asthma

The diagnosis of asthma in children under 3 years of age is difficult since many young children have recurrent episodes of wheezing and cough, typically in response to acute respiratory infections. Moreover, measurement of lung function, airway inflammation and hyperresponsiveness is difficult in this age group. However, some factors indicate a higher risk of persistent respiratory (asthmatic) symptoms beyond young childhood, and children with these characteristics typically progress to *real* asthma, which is less related to respiratory infections and which occurs in between infections and in response to a variety of specific and unspecific stimuli. A positive family history of atopic disease, presence of atopic dermatitis and sensitization to food and aeroallergens predict persistent asthma in childhood and in later life (15).

Episodic wheeze occurs in about 30% of all children, while persistent asthma occurs in about 10% of all children and 5% of adults, but this varies greatly across geographic regions (16). Asthma is more common in young boys than young girls; however, girls are more frequently affected in adolescence and adulthood (17). Atopy is present in about 75% of all children with asthma but only in 50%, or even less, of adults (18). Asthma patients are commonly sensitized to indoor allergens, for example, house dust mite, cockroach, and furred pets. Adults with non-atopic asthma have a more unfavourable prognosis compared with adults with atopic asthma in terms of decline in lung function and persistency of symptoms (19). Concomitant smoking and undertreatment of these patients increases the risk of chronic obstruction and airflow limitation, and this may lead to development of chronic obstructive pulmonary disease (20).

The other atopic diseases frequently accompany or precede asthma, and about 40% of all children with asthma have a history atopic dermatitis (21). Patients with atopic asthma have or will develop hay fever in more than 80% of the cases, whereas only 30% of patients with non-atopic asthma have hay fever (18).

### Hay fever

Hay fever – allergic rhinoconjunctivitis – is present in about 20% of individuals from Western populations (22). It typically develops in late childhood but is most frequent in subjects aged 20–40 years, after which the incidence gradually declines. In many with hay fever, symptoms diminish in middle and late adulthood. Symptoms occur commonly in response to grass and tree pollen but also in relation to indoor allergens such as house dust mite and furred pets (23). Although most people experience seasonal symptoms, about 25% of all affected individuals have perennial symptoms. Seasonality is closely linked to allergic sensitization, often to outdoor allergens, with perennial symptoms being more common in subjects with non-atopic rhinitis. Non-atopic rhinitis is often more severe and is associated with nasal polyps, sinusitis, and recurrent headache (24).

## Occurrence and risk factors for atopic diseases

Atopic diseases are the most common chronic conditions in childhood. Notably, more than 300 million children have asthma worldwide and the prevalence is still increasing in many countries (25). During the past decades asthma and allergy have reached epidemic proportions in most Western societies. Early 20th-century descriptions of these diseases portray them as rare, affecting only few families. But during the past decades atopic diseases have increased markedly in prevalence not only in most Western countries but also in many developing countries. It is unlikely that our genetic makeup has changed over the past decades; therefore, external factors must be considered when explaining the rapid increase in the prevalence of asthma and allergy (26).

### Worldwide prevalence of asthma and allergy

Secular trends in asthma and allergy have been studied most intensely in Western countries. The rise in disease prevalence was particularly apparent between the 1960s and the 1990s, after which the rise evened out. For example, in Australia the prevalence of asthma in schoolchildren increased from 12.9 to 38.6% between 1982 and 1997, and the prevalence of hay fever increased from 22.5 to 44.0% (27). In Denmark, the prevalence of atopic dermatitis increased from 17.3 to 27.3% among children aged 7–17 years between 1986 and 2001 (28), and the prevalence in children living in Scotland increased from 5.3 to 12.0% between 1964 and 1986 (29). Many developing countries have also seen a marked increase in atopic disease occurrence. For example, in South African adolescents the prevalence of eczema increased from 11.8% in 1995 to 19.4% in 2001 (30).

#### The hygiene hypothesis

The hygiene hypothesis was first formulated in 1989 by *David Strachan*. He argued that the decreased exposure to infections and the resulting *cleaner* environment in Western societies over the past decades has led to a higher prevalence of atopic diseases in the population (31). The hygiene hypothesis was originally based on the observation that the youngest child among siblings has a lower risk of atopic diseases, particularly hay fever and atopic dermatitis, compared with his or her brothers and sisters due to a higher infectious load for that child in the rearing environment. In particular, declining family size, improvements in household amenities, and higher standards of personal cleanliness have reduced the opportunity for cross infection, which may have resulted in more widespread clinical expression of atopic disease over the past decades. Several other observations have helped substantiate this theory. For example, children growing up in a traditional farming environment and who therefore have been exposed to a variety of microflora in animal stabling and via unpasteurised cow's milk are also protected against development of allergic diseases (32).

The hygiene hypothesis finds support in the immunological basis of atopic diseases where the infiltration of eosinophils and excessive IgE production depends on a T helper type 2 (Th2) differentiation of naive T cells, with production of IL-4, IL-5, and IL-13 cytokines, as opposed to the Th1 differentiation, which at the same time is inhibited. In particular, the load of prototypical Th1-stimulating infections, such as *hepatitis A*, *typhus*, and *tuberculosis*, has decreased in the past decades in Western societies along with a concurrent increase in use of antibiotics, and this is thought to contribute to the explanation of the increased prevalence of atopic diseases (33).

### Risk factors for atopic diseases

Many risk factors (and some protective factors) for atopic diseases have been identified. Although most of these concern the development of asthma, some factors are also associated with the development of atopic dermatitis and hay fever. Risk factors must be distinguished from triggering factors, which are elicitors of acute allergic or asthmatic reactions in atopic patients, for example, allergens, tobacco smoke, and occupational agents. Nevertheless, there is an overlap between these since, for example, exposure to tobacco smoke and allergens may also be risk factors for development of allergic disease and asthma in the long run.

#### Familial background and genetic factors

Having a close relative with an atopic disease is one of the most distinct risk factors for one's own development of an atopic disease. A child with one atopic parent has a 25% risk of atopy; a child with two atopic parents has a 50% risk of atopy. Genetic studies have uncovered multiple genes with a possible role in the development of atopic diseases, and the clinical expression of these diseases results from a complex interplay between these many genes and from environmental and developmental factors (34).

#### Allergic sensitization

Sensitization to aeroallergens such as house dust mite, animal dander, or pollen is a strong risk factor for development of atopic diseases, particularly asthma and hay fever. The risk of developing atopic diseases is the highest in childhood; nonetheless, it is also increased among adults. However, whether the sensitization itself is a cause of allergic disease is doubtful and sensitization is probably a marker (epiphenomenon) of an ongoing disease process that ultimately evolves into asthma and/or hay fever.

#### Caesarean section

Caesarean section is a risk factor for subsequent development of asthma in the child (35). Possible explanations for this are an increased primary colonisation of ‘allergy-promoting’ skin bacteria at the expense of a decreased exposure to certain ‘allergy-preventing’ bacteria of the vaginal and gastrointestinal flora and an increased risk of neonatal respiratory distress syndrome possibly increasing the risk of later asthma (36).

#### Paracetamol use

Regular intake of paracetamol (acetaminophen) is a risk factor for asthma development both in children and adults (37). Furthermore, maternal intake of paracetamol during pregnancy increases the risk of asthma in the child (38). Depletion of glutathione in alveolar cells weakens the ability of the host to mitigate oxidative stress produced by reactive oxygen species such as superoxide anions and hydroxyl and peroxyl radicals. This loss of antioxidant defences is speculated to induce a cascade of inflammatory mediator release, epithelial damage, and bronchoconstriction, which may ultimately lead to asthma in susceptible individuals.

#### Tobacco smoke

Maternal smoking during pregnancy and exposure to parental smoking in the first years of life increase the risk of asthma symptoms in the child, whereas the risk of atopic sensitization is probably not influenced by exposure to tobacco smoke (39). The risk of asthma is also increased in adulthood among individuals who were exposed to environmental tobacco smoke in early childhood, but this is, at least in part, also due to the adult's own smoking.

#### Respiratory virus infections

Severe infections with respiratory viruses, such as *respiratory syncytial virus*, *human metapneumovirus*, and *rhinovirus*, in the first years of life significantly increase the risk of later asthma (40). Children who suffer from severe bronchiolitis in the first years of life have an up to 50% risk of developing asthma in later childhood compared with an only 10% risk of asthma in children who did not experience such a severe infection. However, this relationship is probably due to the fact that the severe virus infection is a marker of the asthmatic predisposition rather than a direct cause of asthma (41).

#### Obesity

Being overweight or obese increases the risk of asthma, especially adult-onset asthma (42). Some consider a subform of adult-onset asthma mostly in women as part of the metabolic syndrome, with increased body fat, risk of type 2 diabetes and asthma occurring together as a result of shared immunological and inherited factors. Obesity is also a risk factor for uncontrolled asthma and poor response to inhaled corticosteroids (43).

#### Occupational exposures

A large number of occupational agents are associated with outbreaks of asthma symptoms, but some agents are also directly related to asthma development; for example, various types of organic and inorganic dusts, salts, medicinal products, and products from the plastics industry (44). Occupational exposures are a health concern related almost exclusively to adult-onset asthma.

#### Air pollution

Although outdoor air pollution is known to exacerbate pre-existing asthma, it is unclear whether air pollution also plays a causative role in asthma and allergy. Air pollutants may induce airway inflammation and sensitization due to generation of reactive oxygen species. In a study of Swedish children, certain polymorphisms in genes controlling the antioxidative system and inflammatory responses were shown to render children without allergy more susceptible to *de novo* sensitization to aeroallergens when these children were also exposed to elevated levels of traffic nitrogen oxides (45).

#### Diet

Intake of a so-called traditional Mediterranean diet containing high levels of antioxidant and n-3 polyunsaturated fatty acids in the form of olive oil, legumes, fruits, vegetables, unrefined cereals, and wine and a low consumption of meat and processed fat in the form of n-6 polyunsaturated fatty acids is thought to be protective against the development of atopic diseases (46). It is thought that such a diet may lower the risk of asthma and atopy both through the subject's own dietary intake and through the transmission from mother to child during pregnancy.

#### Breastfeeding

There is conflicting evidence concerning the relationship between breast feeding and development of atopic disease, partly because conclusions rest mainly upon observational studies. A recent systematic review found no association between any or exclusive breast feeding and wheezing illness, although there was a high level of heterogeneity between the studies. Particularly, subgroup analysis revealed that any breast feeding slightly lowers the risk of wheeze but slightly increases the risk of asthma defined by specific criteria (47). As to atopic dermatitis, results are likewise heterogeneous but, based on another systematic review, consistent with no strong evidence of a protective effect of exclusive breastfeeding for at least 3 months against atopic dermatitis, even among children with a positive family history (48).

## Conclusion

In spite of great progresses in our understanding of the aetiology and management of asthma and atopic diseases during the past decades, they continue to pose a great burden for patients and society, and only few prophylactic interventions can change their natural history in the long run. The discovery of filaggrin mutations is a new key research area that might hold the key to primary prevention of asthma. Notably, aggressive management of atopic dermatitis may halt the development of asthma and hay fever due to effective maintenance of the skin barrier and a resulting lower risk of percutaneous sensitization to allergens. The clinical effect of such measures awaits evidence from future studies.
